# Complete Genome Sequences of Staphylococcus argenteus TWCC 58113, Which Bears Two Plasmids

**DOI:** 10.1128/MRA.01582-18

**Published:** 2019-04-25

**Authors:** Tohru Miyoshi-Akiyama, Takuma Ohnishi, Masayoshi Shinjoh, Hirotoshi Ohara, Toshinao Kawai, Isamu Kamimaki, Ryo Mizushima, Keisuke Kamada, Yasutomo Itakura, Shigekazu Iguchi, Yutaka Uzawa, Atsushi Yoshida, Ken Kikuchi, Norihiko Takemoto

**Affiliations:** a Pathogenic Microbe Laboratory, Research Institute, National Center for Global Health and Medicine, Shinjuku-ku, Tokyo, Japan; b Department of Pediatrics, National Hospital Organization Saitama National Hospital, Wako-shi, Saitama, Japan; c Department of Pediatrics, Keio University School of Medicine, Shinjuku-ku, Tokyo, Japan; d Department of Plastic Surgery, National Hospital Organization Saitama National Hospital, Wako-shi, Saitama, Japan; e Division of Immunology, National Center for Child Health and Development, Setagaya-ku, Tokyo, Japan; f Department of Infectious Diseases, Tokyo Women’s Medical University, Shinjuku-ku, Tokyo, Japan; Indiana University, Bloomington

## Abstract

Staphylococcus argenteus TWCC 58113 was isolated from a specimen from a 12-year-old boy with purulent lymphadenitis. The S. argenteus TWCC 58113 genome was completely sequenced. The TWCC 58113 chromosome was 2,761,442 bp in size with a GC content of 32.44%. S. argenteus TWCC 58113 was found to harbor two plasmids.

## Abstract (precis)

Staphylococcus argenteus TWCC 58113 was isolated from a specimen from a 12-year-old boy with purulent lymphadenitis. The S. argenteus TWCC 58113 genome was completely sequenced.

## ANNOUNCEMENT

Staphylococcus argenteus is a novel species of coagulase-positive staphylococci that was first distinguished taxonomically from Staphylococcus aureus in 2014 (1). We recently described a 12-year-old Japanese boy with purulent lymphadenitis caused by S. argenteus; to our knowledge, this was the first case of S. argenteus infection in Japan (2). This S. argenteus strain, which belonged to sequence type 2250 (ST2250) in the multilocus sequence type (MLST) scheme for S. aureus (2), was named TWCC 58113. Detailed isolation and growth conditions are described in reference 2. This study reports the complete genome sequences of S. argenteus TWCC 58113, including those for its chromosome and two plasmids, 37.3 kbp and 73.9 kbp. The complete genome of S. argenteus TWCC 58113, which was isolated using a QIAamp blood and tissue kit (Qiagen), was sequenced using Oxford Nanopore technology and the Illumina platform with a flow cell R9.5 system and with MiSeq and Nextera XT systems, respectively. Approximately 7 Gbp of Nanopore data and 2,243,974 paired-end reads of Illumina data were used for genome assembly. Genome *de novo* assembly was performed using SPAdes 3.11.1 (3) in hybrid, plasmid, and careful modes, resulting in three contigs, which corresponded to a chromosome and two plasmids, respectively. This hybrid assembly approach using data from both platforms was highly effective, allowing correct detection of the two plasmids and determining the complete chromosome sequence. After protein-encoding DNA sequence (CDS) extraction using Glimmer 3.02 (4), annotation was performed using NCBI-BLAST 2.1.18+.

**FIG 1:**
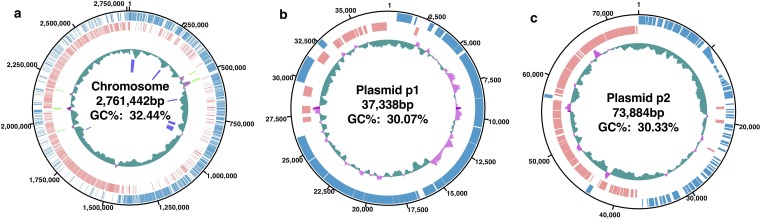
Circular representation of the genome of S. argenteus TWCC 58113 replicons (a to c). Circle 1 (outermost circle) shows distances from the putative origin of replication. Circle 2 shows annotated CDSs encoded on the forward (light blue) and reverse (pink) strands. Circle 3 (innermost circle) shows the GC content, with those greater and lower than the average indicated in green and pink, respectively. (a) The *rrs* operons and genomic islands in the chromosome are also indicated in green and blue, respectively.

The chromosome of TWCC 58113 was 2,761,442 bp with a GC content of 32.44%. This chromosome contained 2,538 predicted CDSs; of these, 2,463 (96.7%) were similar to staphylococcal CDSs, as shown by tblastn comparisons of each CDS in TWCC 58113 with those in the nucleotides of S. aureus N315 (parameters: E value, 0.1; word size, 3; resulting E value range, 0.08 to 1.32 E-180 or 0.00). S. argenteus TWCC 58113 was found to harbor two plasmids; one, designated p1, was 37,338 bp, and the other, designated p2, was 73,884 bp in size (Fig. 1). Comparative analysis of 113 S. argenteus strains, including TWCC 58113, showed that ST2250 strains harbor a significantly higher mean ± standard deviation (SD) number of clustered regularly interspaced short palindromic repeats (CRISPRs) than strains of other STs (2.31 ± 0.67 versus 0.56 ± 0.58; *P* < 0.001). The genome of TWCC 58113 did not contain any intact prophages. Among the 113 strains, TWCC 58113 was the only strain carrying a gene encoding toxic shock syndrome toxin 1 (TSST-1), with this gene being located in a genomic island containing staphylococcal enterotoxins. Analysis of plasmid p2 by Megablast showed that only a 110-bp portion of the 73,884-bp nucleotide sequence was identical to known nucleotide sequences. The CDS alignments of p2 suggested symmetry around the predicted origin of replication (Fig. 1). A blastn search for the predicted origin of replication (positions 1 to 2000) of p2 showed some identity with that of staphylococcal plasmids, including that of Staphylococcus agnetis strain 908 (GenBank accession number CP009624; query coverage, 58%; identity, 70%), suggesting that these share an origin.

### Data availability.

The raw sequence data and chromosome and plasmid sequences have been registered at the DNA Data Bank of Japan (DDBJ) and GenBank under the accession numbers DRA007643, AP018562, AP018563, and AP018564, respectively.
